# Cyclotrimerization Polymers as Precursors for Tailored Porous Carbons and Application in Supercapacitors

**DOI:** 10.1002/open.202500612

**Published:** 2026-04-16

**Authors:** Aleena Jose, Anjana Aravind, Konstantinos Papadopoulos, Kai Konowski, Julia Grothe, Eike Brunner, Stefan Kaskel

**Affiliations:** ^1^ Chemistry and Food Chemistry Inorganic Chemistry I Technische Universität Dresden Dresden Germany; ^2^ Chemistry and Food Chemistry Bioanalytical Chemistry Technische Universität Dresden Dresden Germany

**Keywords:** adsorption, EDLC, NMR spectroscopy, polymeric precursor, porous carbon

## Abstract

Porous carbon materials are considered ideal candidates for electric double‐layer capacitor (EDLC) electrodes owing to their high surface area, electrical conductivity, and chemical stability. The use of synthetic polymers as carbon precursors is an advantageous synthesis approach that enables molecular‐level control over doping, porosity, and other properties for enhanced electrochemical performance. Highly conjugated polymeric frameworks obtained via the triple aldol condensation reaction of different diacetyl‐containing compounds: 2,2′‐diacetylbiphenyl, 4,4′‐diacetylbiphenyl, and 1,4‐diacetylbenzene, serve as efficient precursors for carbon materials, giving good yields upon high‐temperature pyrolysis. The three precursors differ in their molecular frameworks and extent of conjugation, allowing an investigation of the relationship between precursor structure, graphitization, and porosity in the resulting carbons. Our results demonstrate that precursor structural effects become increasingly significant at lower carbonization temperatures, where conjugation and framework rigidity appear to play a role in graphitic ordering. At higher temperatures, several factors, including ease of molecular mobility and the release of volatile components, influence graphitization. The porosity of the resulting carbon materials is correlated with the structural characteristics of the precursor polymers, reflecting the differences in their molecular architecture. These findings highlight the importance of precursor design in tailoring porosity, while temperature remains the dominant factor in controlling graphitization.

## Graphical abstract

Aldol condensation of diacetyl groups containing aromatic systems is utilized for the synthesis of conjugated polymeric precursors, which are pyrolyzed to form porous carbons. This precursor‐based approach for carbon material synthesis provides better control over the properties of the resulting carbon materials.

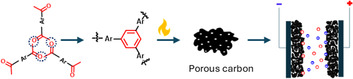

## 1 Introduction

Porous carbon materials have found applications in numerous areas, such as adsorption [1, 2, 3, 4], catalysis [4, 5, 6], and electrochemical energy storage [7, 8, 9]. Their appeal arises from their high porosity, defined pore structures, chemical neutrality, stability, and conductivity. Importantly, these properties can be tailored at the various stages during the synthesis, via raw material/precursor selection [10, 11, 12, 13], precursor modification [14, 15], activation [16, 17, 18] or doping during carbonization [19], and by post‐synthetic treatments [20, 21]. Thus, rational control over precursor chemistry and pyrolysis conditions remains central to designing carbons with application‐specific performance.

Currently, a wide variety of precursors for carbon materials is available. These include biomass‐derived polymers and molecules [22], carbides [23], synthetic polymers [24], aerogels [7], and so on. Biologically sourced polymers, such as lignin and cellulose, and their derivatives are the most popular precursors for carbon materials due to their ease of sourcing and processing [25]. However, their use comes with notable limitations. The molecular structures of natural polymers are largely fixed, offering little opportunity for molecular‐level design. This lack of tunability restricts the ability to engineer porosity or specific carbon nanostructures directly from the precursor. As a result, additional activation steps (physical or chemical) are typically required to enhance porosity or introduce desired surface functionalities [26]. Moreover, biopolymer‐based carbonization often yields lower carbon contents (40%–60%), compared to synthetic polymers, which can achieve yields of up to 70%–80% [27, 28]. The mineral matter and heteroatoms inherently present in natural polymers can also lead to undesired doping, pseudocapacitance, and changes in surface properties, necessitating demineralization or purification processes to obtain chemically uniform carbons [29, 30]. Furthermore, the intrinsic variability of natural feedstocks, depending on origin, processing method, or growth environment, introduces inconsistencies into the final carbon materials [26].

Synthetic polymers, by contrast, offer structural uniformity and tunability. Among them, polyacrylonitrile (PAN) and phenolic resins are widely studied, yielding carbons with controllable porosity and higher carbon yields (up to 70%–80%) [31]. The ability to incorporate heteroatoms or tailor backbone rigidity gives synthetic polymer precursors a distinct advantage in precisely controlling the composition and structure of carbon materials. Within synthetic polymers, conjugated polymers have emerged as especially promising carbon precursors. Their extended π‐conjugation ensures thermal stability and structural retention during carbonization, thereby minimizing the collapse of the framework architecture [32]. Recent advances in conjugated porous networks, including covalent triazine frameworks and conjugated microporous polymers, have further demonstrated molecular‐level control of pore distribution and heteroatom content [33]. Such systems bridge polymer chemistry and carbon science, enabling rational design of carbons with high activity for catalysis, adsorption, and energy storage.

The relationship between precursor framework and carbon properties is well established. Aromatic, carbon‐rich backbones typically yield higher char content and favor graphitic ordering. Crosslinking density, rigidity, and conjugation influence pore formation and nanostructure, while functional groups determine surface chemistry [34]. Temperature, meanwhile, plays a dominant role: higher carbonization temperatures enhance graphitization but reduce heteroatom content and may collapse micropores, while lower temperatures preserve functional groups but produce less conductive carbons [35]. Understanding how precursor structure and temperature interact is therefore crucial for designing carbons with the desired balance of porosity, conductivity, and functionality.

Among synthetic routes, aldol condensation of acetyl‐containing aromatic compounds offers an attractive pathway to conjugated polymer precursors. Triple aldol condensation produces carbon‐rich, thermally stable frameworks with extended conjugation, yielding high carbon content upon pyrolysis. By varying the starting monomer, the molecular architecture and degree of conjugation in the polymer can be controlled, allowing for a systematic exploration of structure–property relationships.

In this work, we focus on polymers synthesized via triple aldol condensation of three different diacetyl compounds: 2,2′‐diacetylbiphenyl (2,2′‐DAB), 4,4′‐diacetylbiphenyl (4,4′‐DAB), and 1,4‐diacetylbenzene (1,4‐DABz) [36, 37]. These precursors differ in framework connectivity and conjugation, providing a platform to investigate how precursor design and carbonization temperature affect porosity and graphitization.

Our study shows that the degree of graphitization in the resulting carbons depends primarily on the pyrolysis temperature, with the precursor structure exerting a limited influence at high temperatures. However, at lower temperatures, differences in conjugation and framework rigidity become more significant in promoting graphitic ordering. In contrast, porosity mirrors the structural features of the precursor polymers, highlighting the role of molecular architecture in controlling pore structure.

These findings emphasize the complementary roles of precursor design and thermal treatment: while temperature dominates graphitization, precursor structure governs porosity. Understanding this interplay provides valuable insights for tailoring polymer‐derived carbons at low synthesis temperatures for applications such as electric double‐layer capacitors (EDLCs), where both conductivity and accessible surface area are critical factors.

## 2 Results and Discussion

### 2.1 Synthesis and Characterization of 2,2′‐DAB

Among the three monomers used for precursor synthesis, 4,4′‐DAB and 1,4‐DABz were available commercially. 2,2′‐DAB was synthesized via Ullmann coupling reaction of 2‐(Iodophenyl) ethan‐1‐one as reported by Che et al. [36] 2,2′‐DAB could be recrystallized as yellowish rhombohedral crystals from the rota‐evaporated dark brown‐colored solution when kept undisturbed for about 48 h. This way, the yield of the reaction could also be improved to 79%.

**FIGURE 1:**
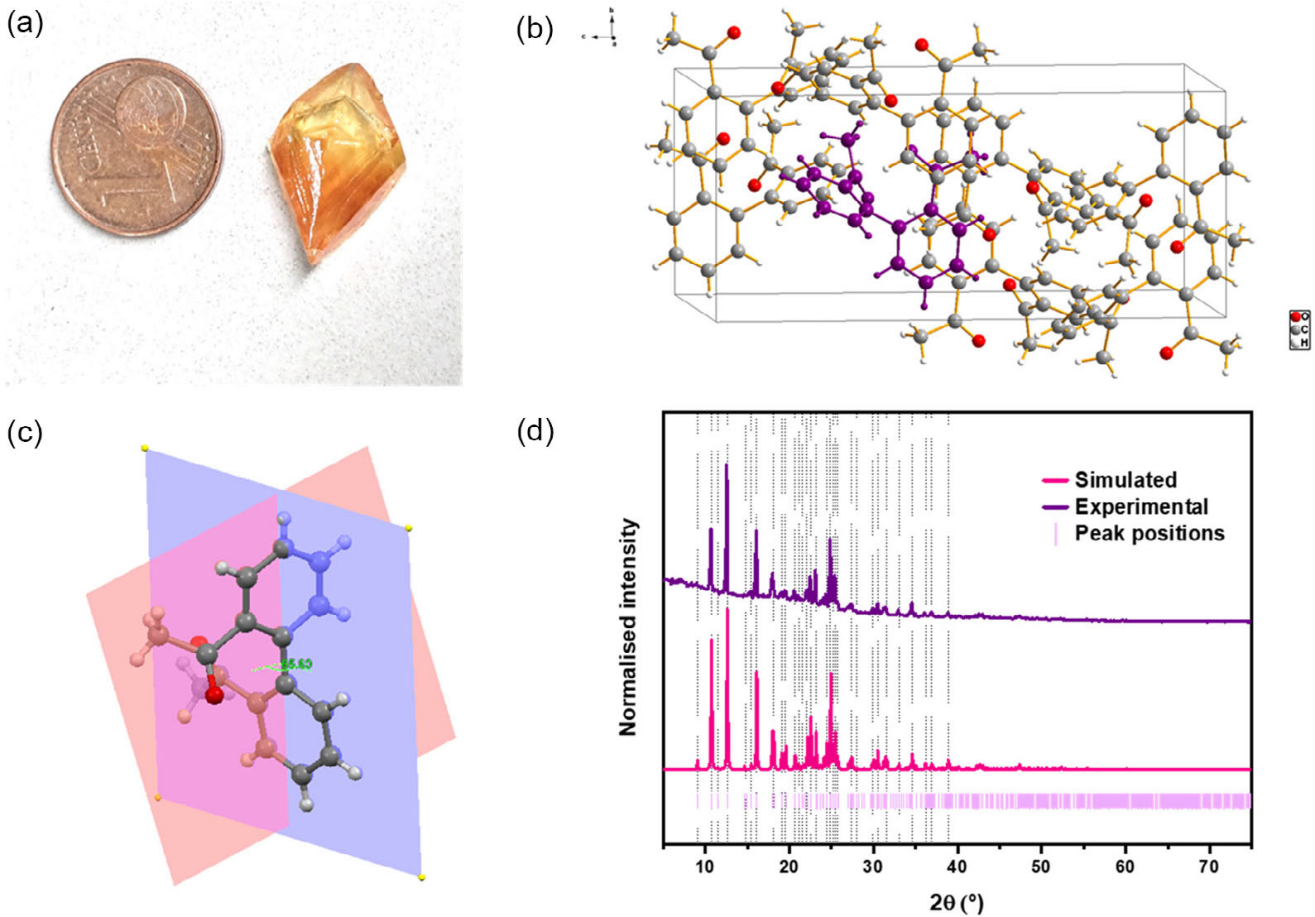
(a) Macroscopic crystal of 2,2′‐DAB obtained after purification; (b) unit cell of 2,2′‐DAB showing orthorhombic crystal system with one 2,2′‐DAB molecule highlighted in purple; (c) molecular geometry of 2,2′‐DAB showing twisted biphenyl rings; (d) simulated PXRD pattern of 2,2′‐DAB against the measured PXRD pattern.

The crystals of 2,2′‐DAB were macroscopic (Figure 1a), and the structure could be determined using single‐crystal X‐ray diffraction. The compound crystallizes in an orthorhombic crystal system with *Pbca*(*61*) space group. The unit cell of 2,2′‐DAB shown in Figure 1b,c shows the molecular geometry of 2,2′‐DAB, highlighting the twisted biphenyl rings in the solid state with a 65.8° angle between the two biphenyl ring planes. The powder XRD pattern of the 2,2′‐DAB crystals matches well with the simulated powder pattern from the SCXRD structure (Figure 1d). The purity was also confirmed by NMR and IR (Figures S1,S2).

### 2.2 Synthesis and Characterization of the Precursor Polymers

**FIGURE 2:**
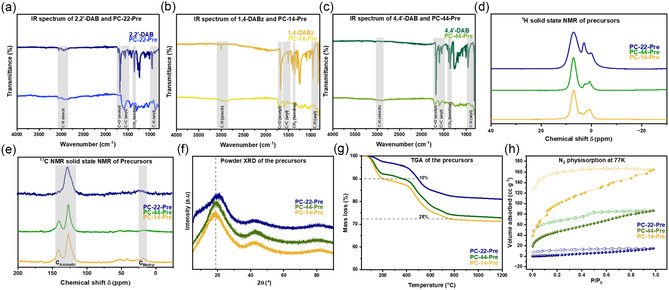
(a–c) The IR spectra of the starting monomers—(a) 2,2′‐DAB, (b) 1,4‐DABz, and (c) 4,4′‐DAB, against the respective precursor polymers synthesized from them—PC‐22‐Pre, PC‐14‐Pre, PC‐44‐Pre; (d) ^1^H solid‐state NMR of the precursors; (e) ^13^C solid‐state NMR of the precursors; (f) PXRD patterns of the three precursors; (g) thermograms of the three precursors in Ar atmosphere; (h) N_2_ physisorption isotherms at 77 K of the three precursors.

**SCHEME 1:**
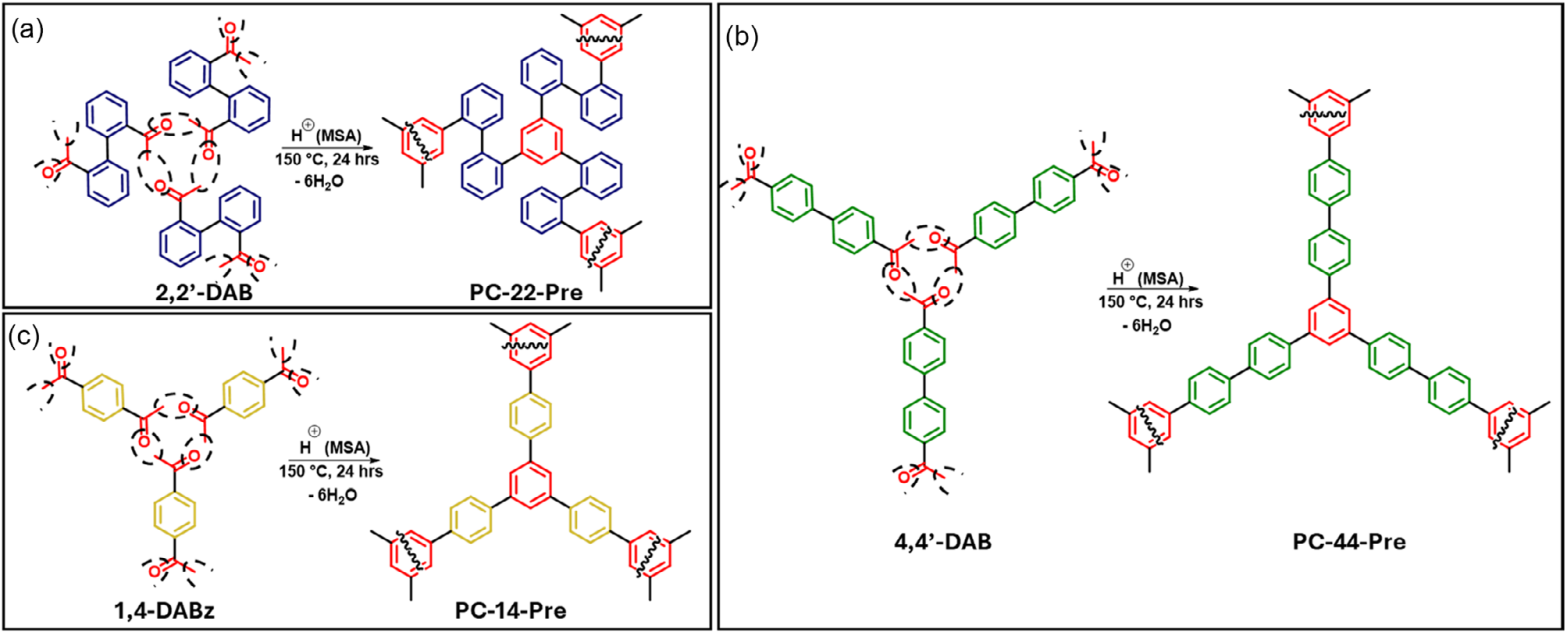
Triple aldol condensation reactions of the three starting monomers: (a) 2,2′‐DAB, (b) 4,4′‐DAB, (c) 1,4‐DABz, yielding precursor polymers PC‐22‐Pre, PC‐44‐Pre, and PC‐14‐Pre, respectively.

The proposed triple aldol condensation reaction scheme for the three starting materials: 2,2′‐DAB, 4,4′‐DAB, and 1,4‐DABz is shown in Scheme 1, giving the polymeric precursors: PC‐22‐Pre, PC‐44‐Pre, and PC‐14‐Pre, respectively. The precursors were characterized using IR spectroscopy. Figure 2a–c presents the IR spectra of the precursors together with those of their corresponding starting materials. The spectra show a clear decrease in the intensity of the carbonyl C=O stretching band at ∼1670 cm^−1^, confirming successful condensation reaction while indicating the presence of some remaining acetyl groups. These residual acetyl signals likely originate from edge sites or incomplete polymerization. In addition the precursor spectra show weakened C—H stretching and CH_3_ bending bands at ∼2900 and ∼1300 cm^−1^, respectively, consistent with the reduced contribution of unreacted methyl groups. The solid‐state ^1^H MAS NMR spectra of the three precursors display a broad resonance, characteristic of rigid, highly conjugated aromatic frameworks (Figure 2d). The dominant intensity arises from aromatic protons distributed throughout the extended π‐systems. Two types of hydrogen atoms are present: aromatic protons (∼7–9 ppm) and methyl protons. However, the methyl protons do not appear as one distinct peak. Instead, several overlapping signals between 0.5 and 3 ppm appear, which indicate structurally slightly different environments. It is possible that part of the methyl groups is shifted due to ring‐current effects exerted by the large aromatic regions, shifting the methyl ^1^H signals to lower chemical shifts. The ^13^C CPMAS NMR spectra of the three precursors exhibit resonances mainly within the aromatic region (125–155 ppm), consistent with their fully conjugated molecular structures (Figure 2e). PC‐22‐Pre presents a single broad aromatic carbon signal, characteristic of the mainly aromatic framework with many similar carbon environments, broadened eventually by variations in local bond state. In contrast, PC‐44 and PC‐14 display narrower, partially resolved signals within the same chemical shift region, reflecting aromatic C—H at ∼125 ppm and fully substituted aromatic carbons at ∼140 ppm. In addition, relatively weak signals (approximately 20 ppm) in the aliphatic region occur, which can be attributed to the aforementioned methyl groups. The weak signals at ∼170 and ∼50 ppm found in the precursors are arising from the carbonyl groups and other hemiacetal‐like side products from incomplete condensation reaction of acetyl groups. ^1^H‐^1^H back‐to‐back (BABA) correlation experiments [38] were performed to examine the spatial proximity of molecular structure by generating double‐quantum (DQ) coherences between protons (Figure S3). In the three spectra, a prominent signal is observed in the aromatic region (∼δ_H_ 6.5–8.5 ppm). Aliphatic signals at ∼δ_H_ 1–3 ppm can be assigned to the methyl protons with slightly different local environments; the signals from PC‐22‐Pre are more dispersed due to stronger ring‐current effects. Furthermore, there is also a correlation between these aromatic signals and aliphatic methyl signals, which is due to the direct attachment of the aliphatic groups to aromatic rings. Heteronuclear correlation (HETCOR) NMR experiments were performed to clarify interactions between carbon atoms and nearby ^1^H nuclei in PC‐44‐Pre and PC‐14‐Pre (Figure S4). PC‐22‐Pre showed very low signal‐to‐noise in the ^1^H‐^13^C cross‐polarization experiment. Moreover, the ^13^C NMR signals are broader than those of the other two precursors, PC‐44‐Pre and PC‐14‐Pre (see Figure 2e). For these reasons, no ^1^H‐^13^C HETCOR spectrum could be recorded within a reasonable measurement time. For PC‐44‐Pre and PC‐14‐Pre, the spectra display well‐defined cross‐peaks in the aromatic region (^13^C ∼120–150 ppm; ^1^H ∼6–9 ppm), consistent with the fully conjugated polyphenylene frameworks of these precursors. Cross‐peaks are observed for both aromatic and methyl proton signals. The methyl ^1^H resonances in the low‐ppm region (∼0.5–3 ppm) show clear correlations with aromatic ^13^C sites (125–150 ppm), confirming that these methyl groups are directly connected to the aromatic framework. The aromatic ^1^H signals also correlate with the same aromatic ^13^C region, demonstrating that both proton environments originate from the expected structural features of the molecules.

The PXRD pattern (Figure 2f), of the precursors shows mainly two signals for the (002) peak and a merged signal for the (100) and (101) reflexes at around 2θ = 20° and 43°, respectively. The first peak is around 2θ = 20°, indicates a nanoporous conjugated structure [39]. The (002) reflex shifted to lower angles than that in graphitic structures due to a higher interlayer distance. The (002) reflex for PC‐22‐Pre is shifted to higher angles compared to the other two, indicating more closely packed layers, as is evident from the structure of the precursor, where the interlayer interactions are better in PC‐22‐Pre [40]. The thermogravimetric analysis performed on the precursors in an Ar atmosphere reveals a two‐step decomposition process (Figure 2g). The first step, occurring below 200°C, indicates the escape of volatile components, and the second step, starting at approximately 400°C, marks the onset of carbonization. The precursors attain a plateau around 600°C, with the PC‐22‐Pre yielding the highest residual mass among the three. From the precursor structures, we observe that PC‐22‐Pre features benzene rings at the ortho positions, promoting cyclization reactions via dehydrogenation, resulting in a more conjugated and less porous carbon framework than the other two precursors. The 77 K nitrogen adsorption isotherms also give the same picture, with PC‐22‐Pre having the lowest porosity (Figure 2h). The kinetic limitation for the N_2_ probe to adsorb in the very narrow pores of the precursors causes the ‘open hysteresis’ leading to the deviation in adsorption and desorption branches of the isotherms [41]. The uptake is lower than expected for PC‐44‐Pre, but this could be due to the blocking of the pores, which later open when pyrolyzed.

### 2.3 Porous Carbons Derived from the Precursor Polymers

The precursors were pyrolyzed at three temperatures—600, 700, and 800°C, as it was observed from thermogravimetric analysis that the mass loss stabilizes at around 600°C for all three precursors. The carbon yield percentages from the three precursors after the three different pyrolyzes are given in Table 1. The PC‐22‐Pre consistently gives the highest yield, as it forms a tightly conjugated network via cyclization reactions, leading to fewer volatile groups that would be eliminated during pyrolysis. The yield also decreases with increased temperatures, as expected.

**TABLE 1:** Yields after carbonization of the three precursors at 600, 700, and 800°C temperatures.

Sample	Yield after 600°C pyrolysis	Yield after 700°C pyrolysis	Yield after 800°C pyrolysis
PC‐22‐	84.7%	83.7%	78.9%
PC‐44‐	79.2%	77.7%	74.4%
PC‐14‐	78.6%	74.8%	75.2%

**FIGURE 3:**
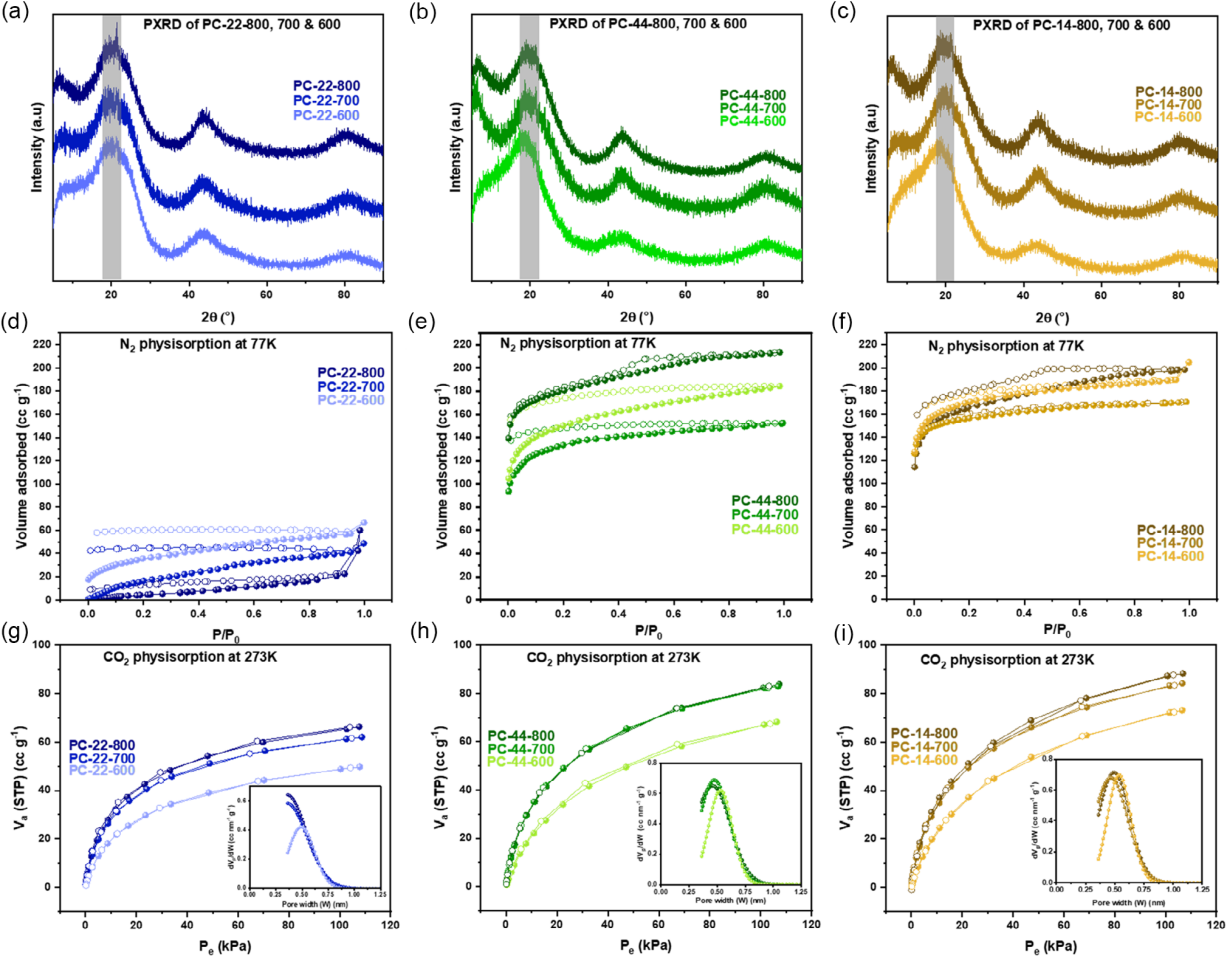
**(**a–c) PXRDs of the porous carbons (a) PC‐22‐800, ‐700, and ‐600, (b) PC‐44‐800, ‐700, and ‐600 and (c) PC‐14‐800, ‐700, and ‐600; (d–f) N_2_ physisorption isotherms at 77 K for the porous carbons (d) PC‐22‐800, ‐700, and ‐600, (e) PC‐44‐800, ‐700, and ‐600 and (f) PC‐14‐800, ‐700, and ‐600; (g–i) CO_2_ physisorption isotherms at 273 K with the inset showing the pore size distribution for the porous carbons (g) PC‐22‐800, ‐700, and ‐600, (h) PC‐44‐800, ‐700, and ‐600 and (i) PC‐14‐800, ‐700, and ‐600.

In the PXRD pattern of the carbon materials, reflexes at around 2θ of 20° for the (002) plane and the (100) and (101) reflexes at around 2θ = 44° were observed, indicating nanoporous turbostratic carbon structures (Figure 3a–c) [40]. The (100) and (101) reflexes are more pronounced with increasing pyrolysis temperatures, indicating horizontal growth of the carbon layer plane [42]. The (002) peak also shifts to higher 2θ angles with increasing pyrolysis temperatures. This indicates that with increasing pyrolysis temperature, the structure has more closely packed benzene rings, thereby improving interplanar interactions and resulting in a lower interlayer distance, which is reflected in a right shift in the PXRD with increasing temperature. The Raman spectra of the porous carbons show the characteristic D and G bands of the sp^2^‐hybridized graphitic structures at around 1350 cm^−1^ and 1590 cm^−1^, respectively (Figure S5). The intensity of the D‐band increases with increasing pyrolysis temperature for the PC‐22‐ and PC‐44‐carbons, indicating that with increasing pyrolysis temperature, the carbon framework undergoes aromatization, leading to the formation of new aromatic domains with more edge sites and structural irregularities, producing stronger defect‐associated vibrations [43]. The IR spectra also show only weak signals for the C—H aromatic stretching from the end groups (Figure S6).

The 77 K N_2_ adsorption isotherms shown in Figure 3d–f show good uptake for the PC‐44‐ and PC‐14‐carbon samples with pore volumes in the range of 0.24–0.33 cc g^−1^. The uptake is low for the PC‐22‐Pre‐derived carbons, as expected from the precursor structure. The pore volumes of PC‐22‐carbons are in the range of 0.08 – 0.1 cc g^−1^. The narrow pore sizes are causing the divergence of adsorption and desorption branches in the carbon materials, so the pore size distribution analysis could not be performed, as at least one branch is not in equilibrium. Hence, the CO_2_ adsorption isotherms at 273 K were also performed to assess the microporosity of the samples (Figure 3g–i) [44]. The micropore volumes derived from the CO_2_ adsorption isotherms are closer in values for the three sets of carbon samples than the pore volumes derived from N_2_ at 77 K physisorption data, indicating that the micropore volume in the samples is similar, but the mesopores are developed only in the carbons from the more porous precursors, namely PC‐14 and PC‐44 (Table 2). The BET surface areas for the samples derived from CO_2_ at 273 K isotherms are almost double or more compared to those derived from the N_2_ at 77 K isotherms, confirming the underestimation of ultramicroporosity with the N_2_ at 77 K isotherm. Another interesting trend is observed in the pore volumes and BET surface areas of the PC‐22 samples as the pyrolysis temperature increases. The specific surface area and pore volumes derived from the N_2_ adsorption data at 77 K decrease with increasing temperature, whereas the microporous specific surface area and the pore volume derived from the CO_2_ adsorption isotherms at 273 K increase. This could be an indication of the macro and mesopores collapsing to form micropores at increasing pyrolysis temperature. Also, even with similar microporosity, the pore size distribution analysis shows pores are around 0.5 nm in width for PC‐44‐ and PC‐14 samples, but are clearly less than 0.4 nm in width for the PC‐22‐700 and PC‐22‐800 samples (Figure 3g–i (inset)). There is no clear trend in the temperature dependence of the porosity of the carbon samples derived from N_2_ adsorption isotherms at 77 K. However, the porosity derived from CO_2_ adsorption isotherms at 273 K increases with increasing temperature, indicating a better development of microporosity as more micropores become exposed at higher pyrolysis temperatures. The porosity to the precursor structure dependency is clear from the above analyses that the porosity of the carbon materials mirrors the structure of their precursors.

**TABLE 2:** Pore volume and BET surface areas of the porous carbons derived from N_2_ at 77 K and CO_2_ at 273 K physisorption experiments.

	Sample	600°C pyrolysis	700°C pyrolysis	800°C pyrolysis
Pore volume (cc g^−1^) From N_2_ at 77 K	PC‐22‐	0.10	0.08	0.09
	PC‐44‐	0.28	0.24	0.33
	PC‐14‐	0.32	0.26	0.31
Pore volume (cc g^−1^) From CO_2_ at 273 K	PC‐22‐	0.06	0.08	0.08
	PC‐44‐	0.08	0.10	0.11
	PC‐14‐	0.09	0.11	0.11
Micropore BET surface area (m^2^ g^−1^) From N_2_ at 77 K	PC‐22‐	129.00	71.65	22.01
	PC‐44‐	563.54	506.80	692.64
	PC‐14‐	645.77	612.13	629.58
Micropore BET surface area (m^2^ g^−1^) From CO_2_ at 273 K	PC‐22‐	673.23	839.22	897.67
	PC‐44‐	924.11	1122.80	1134.50
	PC‐14‐	1198.80	1140.80	1196.00

**FIGURE 4:**
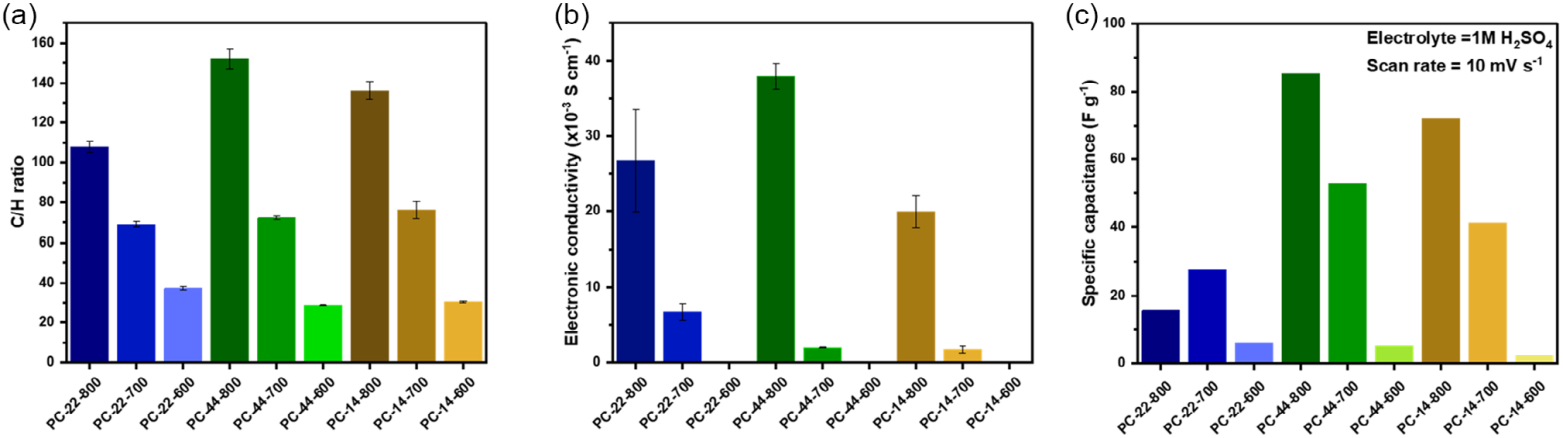
(a) C/H ratio of the porous carbons derived from the CHNS analysis; (b) electronic conductivity of the porous carbon thin films measured using four‐probe method; (c) specific capacitance at 10 mV s^−1^ scan rate of the EDLC systems with porous carbon thin film electrodes with 1 M H_2_SO_4_ as electrolyte.

The elemental analysis was performed to investigate the C/H ratio in the carbon samples and estimate the degree of graphitization in the samples (Figure 4a). The C/H ratio increased with increasing temperature, with PC‐44‐800 having the highest C/H ratio, followed by PC‐14‐800 and PC‐22‐800, respectively. The C/H ratio at higher temperatures does not follow the expected pattern from the structure of the carbon precursors. However, at 600°C, the C/H ratio of the samples is as expected, with PC‐22‐600 having the highest value, followed by PC‐14‐600 and PC‐44‐600. As benzene rings are more closely placed, there are more possibilities for cyclization reactions via dehydrogenation. Hence, it can be observed that the influence of the precursor structure on the amount of conjugation in the final carbon structure is more evident at lower temperatures.

The four‐probe sheet resistance was measured on thin films made from the carbon powder samples, and the conductivity of the samples was calculated using the thin film conductivity equation (SI). Since conductivity is measured on thin films made from powder samples, the conductivity values are also affected by interparticle electron conduction, boundary effects, and other factors. The values are summarized in Figure 4b. The samples pyrolyzed at 600°C were found to have a resistance above 50 MΩ and, therefore, to be nonconducting. At 700°C, PC‐22‐700 has the highest conductivity. In contrast, at 800°C, PC‐44‐800 exhibits the highest conductivity, followed by PC‐22‐800 and PC‐14‐800. The higher thin‐film electronic conductivity of PC‐22‐700 is yet another indication of the higher degree of graphitization for PC‐22‐Pre at lower temperatures compared to PC‐44 and PC‐14‐Pre, due to its structural characteristics.

### 2.4 EDLC Performance

The capacitive performance of the carbon materials was tested in symmetric EDLC systems, with an aqueous electrolyte system of 1 M H_2_SO_4_, as shown in Figure S7, and the specific capacitance values are summarized in Figure 4c. With pyrolysis temperatures of 800°C and 700°C, better graphitization, along with high surface areas and wider pore structures, has enabled the PC‐44‐ and PC‐14‐based carbons to achieve higher specific capacitance. The PC‐44‐800 sample gives the highest specific capacitance of 85.22 F g^−1^. For the systems with 600°C pyrolyzed carbon‐based electrodes, the specific capacitance values exhibit a drastic drop, indicating that major graphitization reactions occur above 600°C. Here, the difference in specific capacitance among the three different carbon material‐based systems is significantly less compared to the samples pyrolyzed at 700 and 800°C. Nevertheless, a small benefit is detected for the PC‐22‐600 samples, indicating better graphitization due to the greater extent of conjugation in the PC‐22‐Pre structure. Additionally, PC‐22‐700 outperforms PC‐22‐800; this may be due to the collapse of pore structures at higher temperatures for the PC‐22 samples, resulting in better porosity and graphitization for the PC‐22‐700 among the three PC‐22 samples. The overall trend of the specific capacitance values at 800°C and 700°C aligns with the porosity properties of the carbon materials, indicating that at high temperatures, the conductivity properties of the carbon materials formed are not significantly influenced by the extent of conjugation in the precursor structures.

## 3 Conclusion

We employed aldol condensation‐derived polymers of diacetyl group‐containing aromatic systems as precursors for porous carbons. The precursors were pyrolyzed at three different temperatures in inert conditions to obtain porous carbon materials with high yields. The porous carbons and the precursors were studied for their structural and porosity properties. PC‐44‐ and PC‐14‐carbons, derived from para‐substituted precursors, were more porous than PC‐22‐carbons, derived from a precursor with closely packed benzene rings synthesized from an ortho‐substituted diacetyl. The porosity properties were found to mirror the structural features of the precursors. At lower pyrolysis temperatures, various properties indicate better graphitization for the PC‐22‐carbons. Under these conditions, the conjugation and framework rigidity of the precursors play a more significant role in graphitic ordering. However, as the pyrolysis temperature increases, the structural effects of the precursors on graphitization properties decrease. This is an indication for major graphitization reactions, such as phenyl bond breaking and structural rearrangements, occurring at temperatures above 700°C. The ease of release of volatile components through accessible pore structures is also a key factor affecting the degree of graphitization at higher temperatures. Hence, for the synthesis of graphitic frameworks at lower temperatures, careful design of the precursors is crucial. This work therefore provides insights into key parameters which need to be considered in the design and synthesis of application‐specific porous carbon materials using milder synthesis conditions.

## 4 Experimental Section

### 4.1 Synthesis of 2,2′‐Diacetylbiphenyl

**SCHEME 2:**
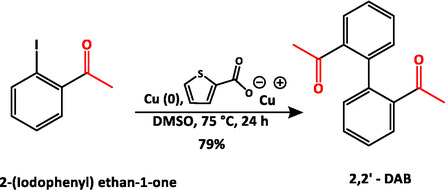
Ullmann coupling reaction of 2‐(iodophenyl)ethan‐1‐one yielding 2,2′‐DAB.

The reaction yielding 2,2′‐DAB is shown in Scheme 2. 25 mmol of 1‐(2‐Iodophenyl) ethan‐1‐one was mixed with 250 mmol of copper powder and 125 mmol of copper‐1‐thiophene carboxylate in 250 ml dimethyl sulfoxide and stirred at 80°C under inert conditions for 24 h. The resulting reaction mixture was filtered to remove the solid catalyst residues, and then the product was isolated by solvent extraction using dichloromethane and ammonia in water solution. The organic phase was collected and dried by extraction over saturated salt solution. The solvent was then removed using a rotary evaporator. The resulting liquid, when kept undisturbed for over 24 h, resulted in crystallization of 2,2′‐DAB crystals from the solution.

### 4.2 Synthesis of Porous Carbon Precursors

Polymeric carbon precursors were synthesized by the aldol condensation reaction on the three diacetyl molecules—2,2′‐DAB, 4,4′‐DAB, and 1,4‐DABz (Scheme 1). The 4,4′‐DAB and 1,4‐DAB were purchased from TCI chemicals and used without further purification. 500 mg of the monomers was dissolved in 3 mL methane sulfonic acid by ultrasonication. Then the solution was heated at 150°C for 24 h for it to undergo condensation reaction. The product was collected by vacuum filtration and was washed multiple times with methanol. The product was further purified by Soxhlet extraction in THF for 24 h to remove unreacted starting materials.

### 4.3 Carbonization of the Precursors

The precursors were carbonized to produce the porous carbons at three different temperatures—600, 700, and 800°C in a tube furnace model—Nabertherm RHTC 80/450/16. The precursors were heated at a constant ramping rate of 2.5°C/min and were held at the target pyrolysis temperature for 3 h. The carbonizations were performed under argon atmosphere flowing at the rate of 200 sccm.

## Supporting Information

Additional supporting information can be found online in the Supporting Information section. The authors have cited additional references within the Supporting Information [45−48]. **Supporting** **Fig.** **1**: The 1HNMR of 2,2′‐DAB (300 mHz, Chloroform‐d) shows all the expected peaks: δ 7.75‐7.72 (dd, 2H), 7.52‐7.42 (m, 4H), 7.19‐7.16 (dd, 2H), 2.26 (s, 6H). **Supporting Fig. 2**: The infrared transmittance spectrum of 2,2′‐DAB shows the signature peaks of the functional groups like C=O, C=C. **Supporting Fig. 3**: ^1^H‐^1^H BABA spectra of the three compounds PC‐22‐Pre, PC‐44‐Pre, and PC‐14‐Pre. The signals of aromatic (^1^H_ar_) and aliphatic (^1^H_al_) protons are indicated, along with the correlation peaks that prove their spatial proximity. Note that PC‐22‐Pre exhibits relatively broad lines and comparably weak aliphatic signal, which prevents the resolution of the correlation peaks. Instead, the correlation signals occur as shoulders. Dashed grey lines indicate the correlation signals. **Supporting Fig. 4**: ^1^H–^13^C HETCOR experiment of precursors (a) PC‐44‐Pre and (b) PC PC‐14‐Pre with 4 ms contact time. **Supporting Fig. 5**: Raman spectra of a) PC‐22‐800, 700 & 600; b) PC‐44‐800, 700 & 600; c) PC‐14‐800, 700 & 600 showing the D‐ and the G‐bands. **Supporting Fig. 6**: IR spectra of the pyrolyzed samples – a) PC‐22‐800, 700 & 600; b) PC‐44‐800, 700 & 600; c) PC‐14‐800, 700 & 600. **Supporting** **Fig.** **7**: The cyclic voltammograms of EDLCs made from a) PC‐22‐800, 700 & 600; b) PC‐44‐800, 700 & 600; c) PC‐14‐800, 700 & 600 with 1 M H_2_SO_4_ as electrolyte at 10 mV s^–1^ scan rate.

## Funding

This study was supported by Deutsche Forschungsgemeinschaft (GRK 2861–491865171).

## Conflicts of Interest

The authors declare no conflicts of interest.

## Supporting information

Supplementary Material

## Data Availability

Crystallographic data for 2,2′‐DAB (CCDC number 2524479) were deposited at the Cambridge Crystallographic Data Centre and could be obtained free of charge from http://www.ccdc.cam.ac.uk/structures.
