# Supplementary material for: Cyclotrimerization Polymers as Precursors for Tailored Porous Carbons and Application in Supercapacitors
Source: ChemistryOpen. 2026 Apr 16;15(4):e202500612. doi: 10.1002/open.202500612 (PMC13087105; doi:10.1002/open.202500612)
Supplement: Supplementary file 1 — Supplementary Material [file OPEN-15-e202500612-s001.pdf]

Cyclotrimerization Polymers as Precursors for Tailored Porous Carbons and Application in Supercapacitors

Aleena Jose*,[1] Anjana Aravind,[2] Konstantinos Papadopoulos,[1] Kai Konowski,[1] Julia Grothe,[1] Eike Brunner[2] and Stefan Kaskel[1]

[1]Inorganic Chemistry I, Technische Universität Dresden Bergstrasse 66, 01069 Dresden (Germany)
[2]Bioanalytical Chemistry, Technische Universität Dresden Bergstrasse 66, 01069 Dresden (Germany)

Instruments and methods

Powder XRD was recorded using on an Empyrean Alpha-1 powder X-ray diffractometer (Malvern Panalytical) employing Cu Kα_1 radiation.

FTIR spectra were obtained with Bruker Vertex 70 (32 scans, 2 cm^{-1} resolution).

Raman spectroscopy was recorded using a DXR SmartRaman Spectrometer from Thermo Scientific with an excitation laser of wavelength 532 nm.

NMR spectroscopy - Solid-state ^{13}C nuclear magnetic resonance (NMR) spectra were acquired using a Bruker Ascend 300 MHz NMR spectrometer operating at 75.47 MHz for 13C. A commercial double-resonance 4 mm magic-angle spinning (MAS) NMR probe was employed. Around 6 mg samples were packed into rotors and spun at 15 kHz. Cross-polarisation (CP) was applied to all samples at the magic angle. The CP contact time was set to 3 ms, with a recycle delay of 3 s. Free induction decays (FIDs) were collected over 30,000 scans, resulting in a total acquisition time of 25 hours. The spectral width was 30 kHz. Spectra were referenced to tetramethylsilane (TMS), using adamantane as a secondary standard with its signal at 29.5 ppm.

A 2D ^{1}H-^{13}C HETCOR experiment was performed on a Bruker MAS NMR spectrometer operating at 300 MHz. The sample was measured using a MAS probe at a spinning rate of 15 kHz. The direct ^{13}C dimension was acquired with 2048 points, a spectral width of 22.7 kHz, and an acquisition time of 45 ms, while the indirect ^{1}H dimension used 200 t_1 increments with a spectral width of 44.1 kHz. The CP contact time was 0.5 ms, and the recycle delay was 3 ms in the HETCOR experiment.

The ^{1}H MAS and ^{1}H - ^{1}H MAS BABA solid-state NMR spectra were acquired on a Bruker Avance 800 MHz spectrometer. A 1.3 mm double-resonance MAS probe head was used at a MAS frequency of 50 kHz for ^{1}H -^{1}H MAS BABA experiments, whereas a 3.2 mm double-resonance MAS probe head was used at a MAS frequency of 15 kHz for ^{1}H MAS solid-state NMR.

For ^{1}H spectra, 128 scans were collected with a spectral width of 156 kHz and a 3 s delay at a MAS frequency of 15 kHz. For ^{1}H - ^{1}H MAS BABA, the direct ^{1}H dimension was acquired with 8192 points, a spectral width of 156 kHz, and an acquisition time of 26 ms. The indirect ^{1}H dimension was acquired with 128 points, a spectral width of 50 kHz, and an acquisition time of 1.28 ms. The recycle delay was 3 ms, and a total of 96 scans were measured in the 1H-1H BABA experiment. All spectra were referenced to TMS (tetramethylsilane), with adamantane used as a secondary reference at 1.85 ppm for ^{1}H and 29.5 ppm for ^{13}C.

Thermogravimetric analysis - The measurements have been carried out with a Netzsch STA 449 F5 Jupiter from room temperature to 1200 °C with a heating rate of 2 K/ min under an inert argon atmosphere.

Physisorption experiments – Nitrogen physisorption at 77 K was measured using Quantachrome Quadrasorb SI surface area and pore size analyzer, carbon dioxide physisorption at 273 K was performed using Belsorp Max II. The samples were degassed before the measurements at 150 °C for 24 hours. Pore size distribution was calculated from the CO_2 physisorption isotherm at 273 K using non-local density functional theory (NLDFT) with a carbon slit-pore kernel (desorption branch), as implemented in the BelMaster software. The total pore volumes were determined at P/P_0=0.98 from N_2 physisorption isotherms at 77 K and at P/P_0=0.03 from CO_2 physisorption isotherms at 273 K. The surface areas were determined using Brunauer-Emmett-Teller (BET) method in the P/P_0 range of 0.001 – 0.05 with N_2 physisorption isotherms at 77 K and in the P/P_0 range of 10^{-5} – 0.03 with CO_2 physisorption isotherms at 273 K.

Conductivity was calculated using the sheet resistance measured on the carbon thin films using a 2401 SourceMeter from Keithley and a SP4 Signatone Corp Four-point probe head. Free-standing carbon films were prepared by mixing fine carbon powder, obtained by milling bulk carbon at a frequency of 30 s^{-1} for 3 minutes, with PTFE (5 wt%) and rolling it out into thin films. The thickness of the sheets was measured using a thickness gauge, and the measured thickness ranged from 0.03 to 0.05 mm. Hence, the thin film conductivity equation was used:

$$S = \frac{1}{(R_S \times t)}$$

Where S is the conductivity, R_S is the thin film resistance, and t is the thickness of the film.

Elemental analysis was carried out using a Vario MICRO-cube Elemental Analyzer, which is equipped with simultaneous analysis for Carbon, Hydrogen, Nitrogen and Sulphur (CHNS). The samples for the analysis were prepared in glove box after drying them overnight in vacuum oven at 60 °C. The analysis was performed thrice, and the mean value is reported.

Electrochemical characterisation

EDLC cells were made using a custom-built polyether ether ketone (PEEK) cell with spring-loaded titanium pistons. The porous carbon materials (milled at a frequency of 30 s^{-1} for 3 minutes to produce fine powder) were mixed with PTFE as a binder in a weight ratio of 95:5 and rolled out into thin sheets. 10mm diameter electrode discs were cut out from the sheets using a template with weights ranging from 2.5 to 4mg. Whatmann filter paper was used as the separator. Cyclic voltammograms were performed using a VMP3 Potentiostat (BioLogic Science Instruments). The specific capacitance is calculated from the CV using the formula:

$$C = \frac{\int_{U_1}^{U_2} I \, dU}{\Delta U \, \nu \, m}$$

where C is the integral specific capacitance, I is the current response on cycling the voltages from U_1 to U_2 at the scan rate ν, ΔU is the voltage window used, and m is the mass of the electrode.

Crystal data

Single-Crystal X-ray diffraction

Single crystal XRD (SCXRD) data were collected at RT using a Rigaku XtaLAB Synergy-S diffractometer equipped with a microfocus PhotonJet X-ray source (Mo-Kα radiation, λ=71.073 pm) and a hybrid photon counting detector (Eiger2 R1 M CdTe, Dectris). The raw data were processed using CrysAlisPro and automated empirical absorption correction was performed. The structures were solved using SHELXT-2019 [45] and refined by full-matrix least-squares analysis (SHELXL-2019) [46, 47] using the

program package OLEX2 [48]. Non-hydrogen atoms were refined anisotropically, and hydrogen atoms were constrained to ideal geometries and refined with fixed isotropic displacement parameters (in terms of a riding model).

Chemical Formula	$C_{16}H_{14}O_2$
Molecular Weight [g/mol]	238.27
Crystal System	Orthorhombic
Space Group	*Pbca(61)*
Temperature [K]	286
a, b, c [Å]	a=15.3372(6) Å, b=8.6073(3) Å, c=19.5088(8) Å
α, β, γ [°]	90°, 90°, 90°
V [Å^3]	2575.40(16)
Z	8
Density, Calculated [g/cm^3]	1.229
μ/mm^{-1}	0.080
F(000)	1008.0
Crystal size/mm^3	0.065 × 0.056 × 0.033
Radiation Type	Mo Kα, λ = 0.71073 Å
Diffractometer	Four-circle diffractometer, XtaLAB Synergy, Duaflex, Eiger2 1M
Absorption Correction	Multi-scan
2θ range for data collection [°]	4.95 to 59.488
Index ranges	-20 ≤ h ≤ 15, -11 ≤ k ≤ 9, -24 ≤ l ≤ 15
Reflections collected	11545
Independent reflections	3114 [R_{int} = 0.0396, R_{sigma} = 0.0402]
Data/restraints/parameters	3114/0/165
Goodness-of-fit on F^2	1.075
Final R indexes [I>=2σ (I)]	R_1 = 0.0464, wR_2 = 0.1175
Final R indexes [all data]	R_1 = 0.0741, wR_2 = 0.1433
Largest diff. peak/hole / e Å^{-3}	0.19/-0.19

Figures

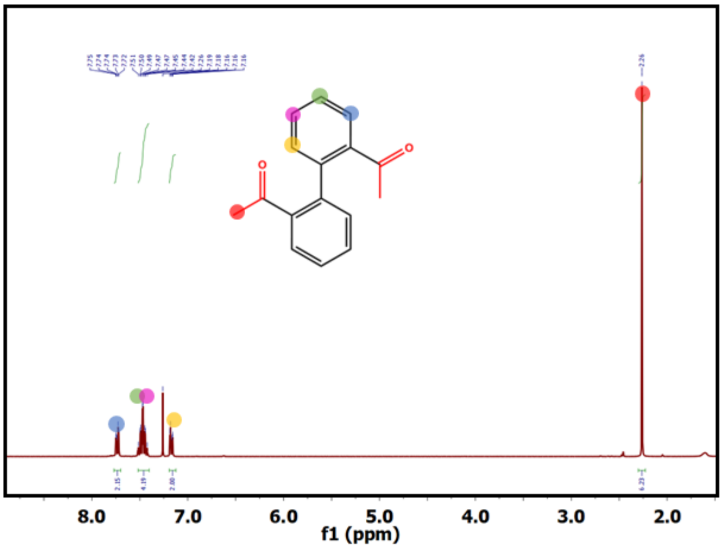

Figure S1: The 1H NMR of 2,2'-DAB (300 mHz, Chloroform-d) shows all the expected peaks: δ 7.75-7.72 (dd, 2H), 7.52-7.42 (m, 4H), 7.19-7.16 (dd, 2H), 2.26 (s, 6H).

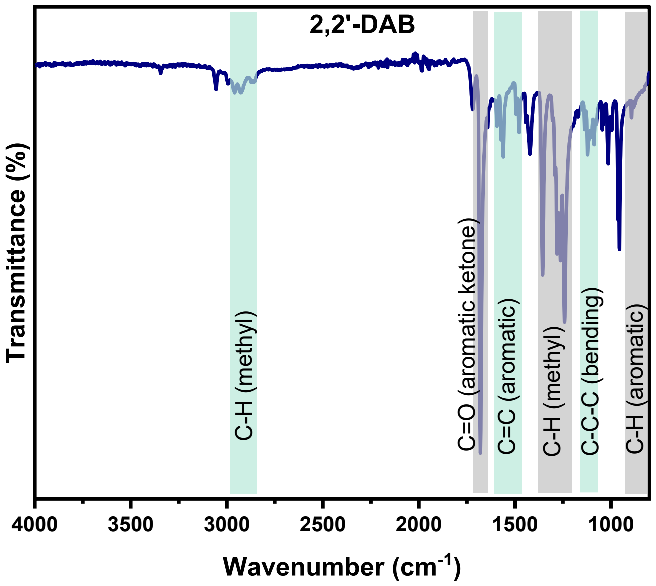

Figure S2: The infrared transmittance spectrum of 2,2'-DAB shows the signature peaks of the functional groups like C=O , C=C.

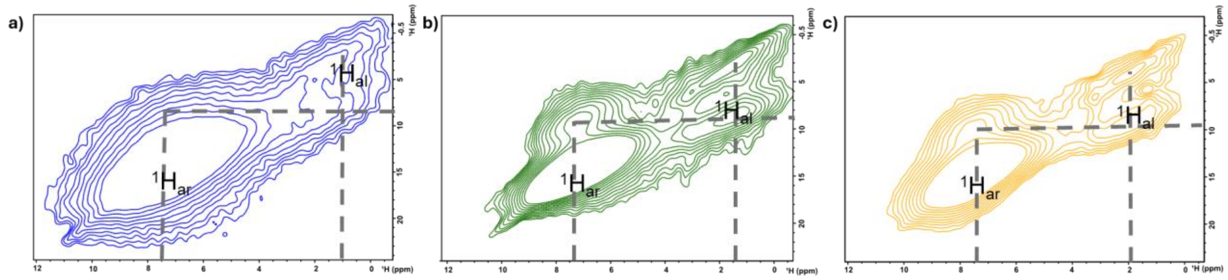

Figure S3. ^{1}H-^{1}H BABA spectra of the three compounds PC-22-Pre, PC-44-Pre, and PC-14-Pre. The signals of aromatic ($^{1}H_{ar}$) and aliphatic ($^{1}H_{al}$) protons are indicated, along with the correlation peaks that prove their spatial proximity. Note that PC-22-Pre exhibits relatively broad lines and comparably weak aliphatic signal, which prevents the resolution of the correlation peaks. Instead, the correlation signals occur as shoulders. Dashed grey lines indicate the correlation signals.

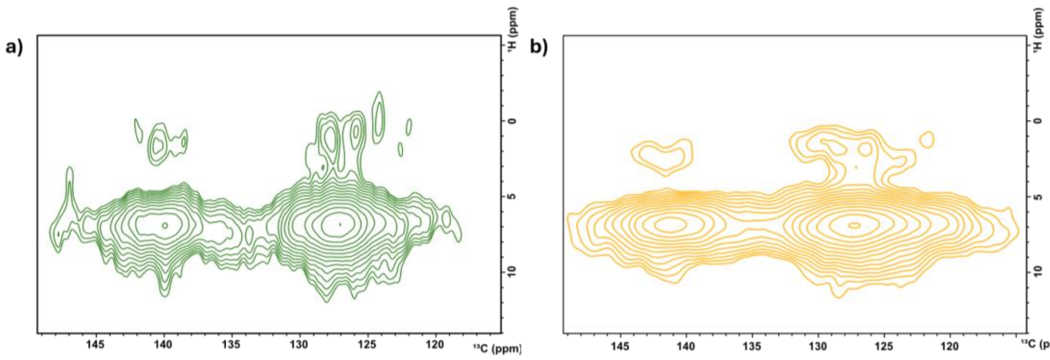

Figure S4. ^{1}H–^{13}C HETCOR experiment of precursors (a) PC-44-Pre and (b) PC PC-14-Pre with 4 ms contact time.

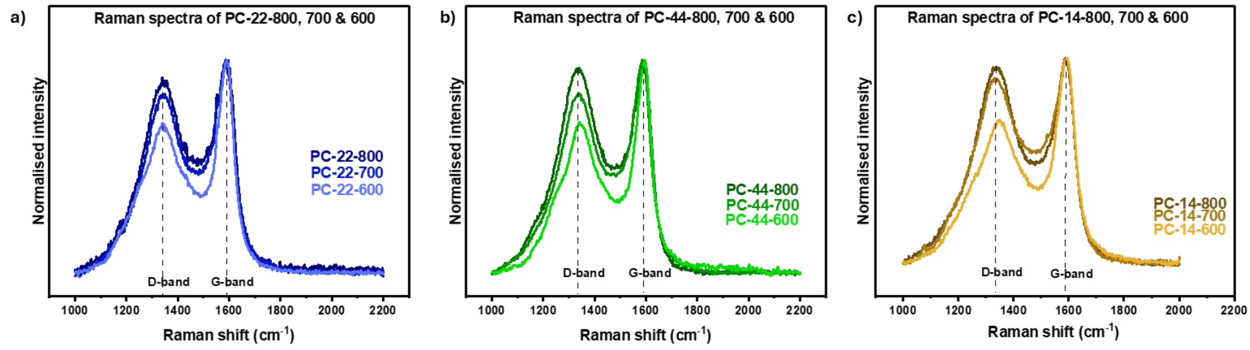

Figure S5: Raman spectra of a) PC-22-800, 700 & 600; b) PC-44-800, 700 & 600; c) PC-14-800, 700 & 600 showing the D- and the G-bands

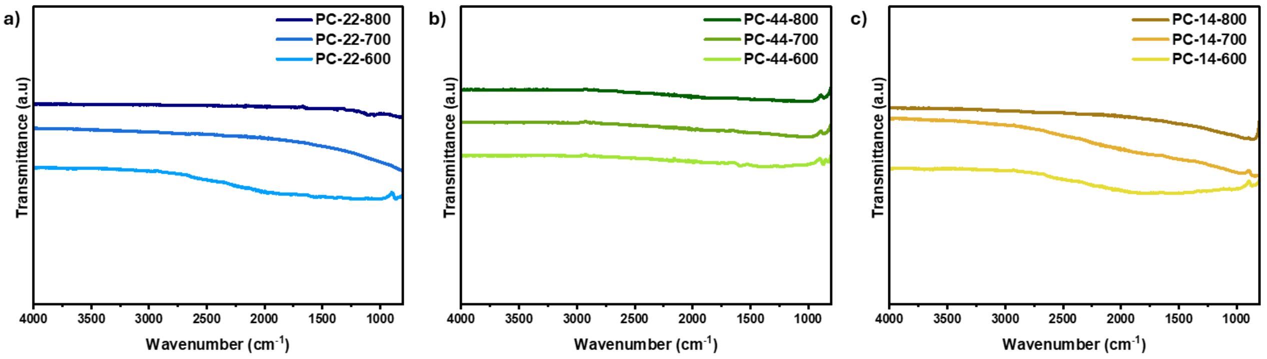

Figure S6: IR spectra of the pyrolyzed samples – a) PC-22-800, 700 & 600; b) PC-44-800, 700 & 600; c) PC-14-800, 700 & 600.

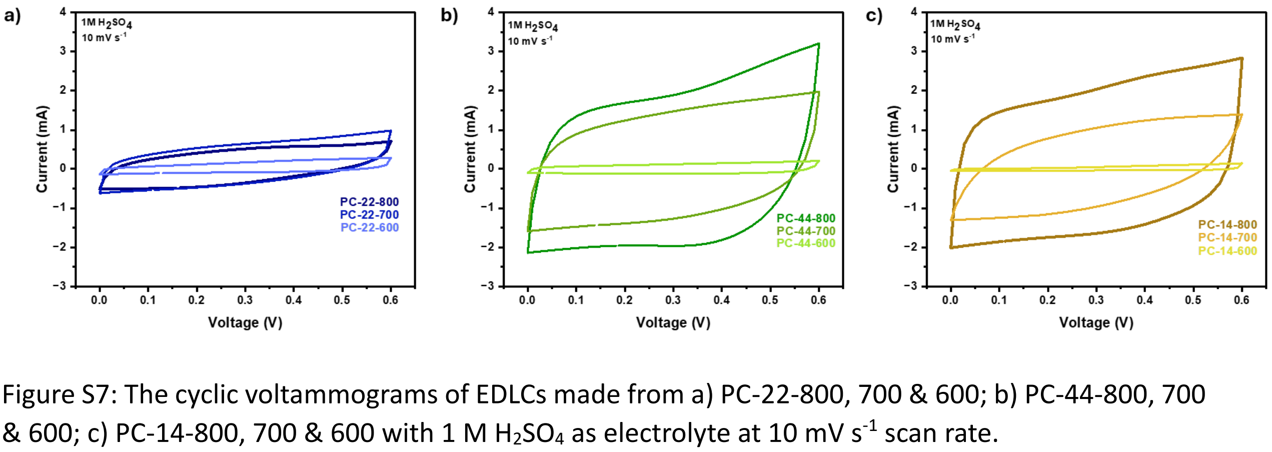

Figure S7: The cyclic voltammograms of EDLCs made from a) PC-22-800, 700 & 600; b) PC-44-800, 700 & 600; c) PC-14-800, 700 & 600 with 1 M H_2SO_4 as electrolyte at 10 mV s^{-1} scan rate.
